# Selections

**Published:** 1817-05

**Authors:** 


### PART III.

### SELECTIONS.

—— Factorum est copia nobis.

### FOREIGN JOURNALS.

I. Physiology.—*Report made to the Institute, by Messrs. Hallé and Pinel on a Memoir of M. Magendie, relative to the Deglutition of Air.**

This Memoir is a continuation of those previously read by M. Magendie on the mechanism of vomiting. In his experiments on vomiting, this physiologist had remarked that the operation was preceded by efforts, during which the stomach of the animal swelled immediately after an act of deglutition, and that this phænomenon preceded vomiting These efforts appeared to him to be the same as those which accompany the nausea commonly felt before vomiting; and hence he presumed that, at this instant, there took place a deglutition of air, which was obviously the cause of the dilatation of the stomach invariably observed under these circumstances.

Thus viewed, the phænomenon appears as one of the conditions by the aid of which vomiting is effected; and it is, moreover, connected with many not less interesting observations attendant on different operations of the animal œconomy.

Several physiologists, among whom are M. Gosse of Geneva, and Magendie, have successfully attempted the deglutition of air, and availed themselves of this mean to provoke vomiting: for thus nausea was induced, and the stomach irritated, till at length it was relieved by the expulsive process.

A young man, with a view of evading the conscription, afforded an example of this faculty possessed to the degree of distending the intestines as well as the stomach, so as to simulate tympanitis with all the apparent suffering of violent disease. The air, thus accumulated, was afterwards discharged by the œsophagus and rectum. It required no small share of talent and attention to detect this singular artifice.

Analogous phænomena are exhibited by several diseases. We have seen like alternations of deglutition and discharge in hysterical affections. Tumefaction of the epigastric region by flatus and

* Rapport fait à l'Institut. par M. M. Hallé et Pinel, &c. Journal de Medicine, par Leroux, Mai, 1816.

eructations, similar to those of hysteria, are very common in hypochondriacal diseases. We have, at present, under observation, an instance of tension of the stomach, followed by rapid and singularly impetuous eructations, in an aged lady, suffering from an affection which disturbs the digestive process and obstructs the passage of the aliment in the duodenum. The known sympathies of the throat with the stomach, and of both with the uterus and the epigastric nervous centre, seem, in fact, to be a common source of flatulence, in many diseases, whether of the alimentary canal, or of a nervous and spasmodic character. But these examples and analogies are to be regarded as indications rather than a direct demonstration of what M. Magendie proposes to explain.

The experiments made by him upon animals, have afforded him, on the objects of his research, the clearest evidence. We have repeated those experiments, the results of which are announced in his memoir; and shall proceed to describe with precision what we have observed. Vomiting is really provoked in animals, either by exciting the external surface of the stomach laid bare, or injecting into the veins an emetic solution, as that of tartrite of antimony. Both these processes have the advantage of not acting directly on the organs of deglutition, and leaving them to obey exclusively the natural movements of these parts when the stomach has just been provoked to vomiting by causes which would be proper to determine it, if the organs, by which it is executed, were completely sound.

The right jugular vein of a young dog was exposed and tied in the middle of the neck. Besides this, the integuments of the abdomen were cut, and the intestines laid bare and separated, in order to disengage the stomach; in which were some fragments of bones previously swallowed by the animal.

On pressing the peritonœal surface of the stomach, about the great curvature, it was observed to swell and fill with air. The animal was seen, at the same time, to make movements of deglutition, preceded by motion of the head anteriorly, similar to those which are made in the efforts attendant on nausea. On examining these efforts, we observed that they were executed in the following manner: The larynx came forward from the vertebral column, was then drawn upwards and forwards towards the jaw; and, lastly, was retracted to its original site. The animal, at the same time, thrust his neck anteriorly, as though to assist these movements; and endeavoured to open the jaws, which had been secured by a muzzle. During these perceptible movements, the stomach was dilated and filled with air; which, on pressure of the distended organ, was afterwards expelled by the mouth. Part of the food previously swallowed was then found near the animal.

The execution of these movements has evidently for its effect the dilatation of the pharynx and superior part of the œsophagus, and consequent augmentation of the volume of contained air. This air is prevented from escaping anteriorly by the application of the base of the tongue to the palate, of the velum palati to the nasal fossæ, and of the epiglottis to the larynx. Thus the air becomes inclosed in a cavity without any other issue than the œsophagus: and this cavity contracting, and, at the same time, executing a movement posteriorly, propels the contained air into the œsophagean tube; in the same manner as it conveys all substances which obey the act of deglutition.

A solution of twelve grains of tartrite of antimony was afterwards injected into the jugular vein below the ligature. In about two minutes, the movements were produced and nausea manifested: then the stomach sensibly swelled and became filled with air which was discharged on pressure.

We may naturally conclude, from these experiments, that the movements attendant on nausea, and preceding the expulsive action of vomiting, are movements of deglutition by which a considerable quantity of air is conveyed into the stomach; that this introduction becomes a condition favourable to the act of vomiting which it is capable of, itself, exciting, and even, as it should seem, independently of the causes otherwise provoking it;* that it is probably this which is observed in spasmodic diseases, particularly hysteria and hypochondriasis, where the throat is often affected with sympathetic spasms, followed by borborygmi, eructations, and singular swellings of the neck and epigastric region; and that, consequently, the phænomenon, analysed and developed by the experiments of M. Magendie, interests, in several respects, the study of the animal œconomy and that of diseases.

*Signed,* G. Cuvier, *Perpetual Secretary.*

---

### II. Pathology.—*Memoir on Inflammation of the Hernial Sac.*†

( Concluded from Page 248. )

*Description of the Disease.*—In every abdominal hernia, properly so called, the parts composing it push before them that portion of peritonæum which covers, and is adjacent to, the natural or adventitious orifice by which they escape. This portion serves as a covering to the displaced parts, and is called the *hernial sac.*

If the hernia be small and recent, the sac may be reduced with it. Otherwise, in consequence of contracting adhesions with the external cellular structure, the sac continues unreduced; and contracts, or is even sometimes completely obliterated to the level of the orifice through which it passes, both by the constriction of the margin of the orifice, and by the pressure of the bandage, upon it.

---

* The experiment of Gosse and others sufficiently demonstrates, that the deglutition of air is a phænomenon which occurs under many other circumstances even without being succeeded by vomiting.

† Bulletin de l'Athénée de Médecine de Paris. *Julliet,* 1816.

To this permanence of the sac may be partly attributed the frequent relapse of hernia, and the successive increase of volume proportionate to its duration. It is the existence of the sac which sometimes disposes to a species of hydrocele disappearing on pressure and in the horizontal posture.

The pad of the truss, applied wholly or in part upon the sac, compresses and even irritates it, and may thus induce chronic or acute inflammation terminating in two different ways: 1. Either the opposite parietes of the sac, brought together by the compression, adhere, and the cavity is partially or completely obliterated. (I have, many times, ascertained this fact by the dissection of subjects of old herniæ long supported by a bandage. Although the peritonæum entered the abdominal ring in the shape of a funnel, I found no other trace of the hernial sac than a thick cellular layer in its place): Or, 2dly, this inflammation augments the wonted exhalation of the internal surface of the sac, and alters its nature. The parietes, separated by the fluid, tumify, become thick and indurated, and implicate the adjacent cellular substance: thus the tumour is developed.

The previous strangulation of the hernia, and improper attempts at reduction, may produce a kind of fluxion on the sac which continues after its return, and is farther aggravated by the pressure of the bandage. In this case, the inflammation may be very acute, and exhibit the characters of phlegmon or bubo.

The causes of inflammation of the sac then are, 1st. Hernia, which by its duration and frequency has rendered the sac permanent: 2dly. The irritation resulting from pressure of the truss-pad, from strangulation, and injudicious trials at reduction; lastly, we may admit the influence of peculiar predisposition in some individuals. This predisposition is observed in many diseases; and may, for instance, explain the cartilaginous alteration of the tunica vaginalis which occurs in some cases of hydrocele.

Since the age, and particularly the frequency, of hernia is the predisponent cause of this morbid state of the hernial sac, the sac of inguinal hernia in the male, and that of femoral hernia in the female, must necessarily be most subject to it. On the other hand, the more superficial site of inguinal hernia exposes its sac to more direct compression. Hence the alteration of this must be more common than that of the femoral sac, more deeply seated and defended from pressure by a thick covering of cellular substance and lymphatic glands.

Hitherto I have clearly observed this alteration only in the male subject. Yet I have no doubt that it may exist also in the female. I have, several times, seen, in consequence of femoral hernia reduced after strangulation, a tumour possessing exactly its site, figure, and relations; but which the absence of symptoms, notwithstanding its irreducibility and compression, induced me to suppose formed by alteration of the sac. It might indeed depend on a portion of adherent omentum. I had not the opportunity of solving these doubts by dissection.

Under the influence of the causes just cited, there appears at the abdominal ring (I take for type the alteration of the sac of *inguinal* hernia) a rounded or pyriform, regular and resistent tumour. The pain, preceding or accompanying it, is not commonly severe, but sometimes becomes so from pressure. Adherent to the ring, it is little if at all moveable. Although very firm, it sinks beneath compression with the finger, and yields the same sense of crepitation which results from pressure of sulphur or powdered starch. On removal of the compression, the tumour immediately resumes its original form, in consequence as well of the contained fluid as of the great elasticity of its parietes. The thickness and density of these render fluctuation at first imperceptible. Left to itself, and not irritated by bandage or compression, this tumour may give rise to no local or general symptoms, as in the subject of the second case. On the contrary, it becomes very painful by irritation. Acute inflammation seizes it, and is successively propagated to the adjacent cellular structure and skin. When yet more severe, may it not traverse the ring, and be communicated to the peritonæum and contiguous viscera? And to such diffusion, ought not the abdominal inflammation of the first case to be referred?

However this be, the product of exhalation of the internal surface of the sac increases and changes more and more. Unable to distend the resisting parietes of the cavity, it corrodes and externally attenuates them, produces an evident fluctuation, and at last escapes by several orifices. These continue fistulous till the parietes of the cavity are nearly destroyed by suppuration; which process requires a long time for its accomplishment, as in the third case.

The fluid, first discharged, is rather puriform than purulent; viscous, flocculent, and altogether resembling the product of inflammation of serous membranes in general, and of the peritonæum in particular. It soon assumes the character of ordinary pus. Notwithstanding the complete vacuity of the tumour, it does not collapse, except momentarily, on pressure; or, when its parietes have been softened or destroyed by suppuration. Thus, it long exhibits a patent cavity, which, at every dressing, is found full of pus. Yet the parietes soften and insensibly waste. Cellular granulations arise, and fill up the bottom of the wound; and thus cicatrization is accomplished.

Ledran states, that a portion of omentum retained in the sac suppurated, and that the fluid, effused into the abdomen by the neck of the sac, destroyed the patient. We may infer from analogy, that in the disease under consideration, the pus, finding less resistance from the neck than from the parietes of the sac, may escape into the abdomen, and induce dangerous consequences. *Observat. Chirurg.* tom. ii. p. 63.

Such are the characters, symptoms, progress, and termination of the disease, when simple; but complications may exist, rendering its diagnosis difficult, and issue fatal. 1st. The alteration of the sac may exist in a person of cancerous disposition. The sac, when irritated, will then become the centre of cancerous action, and exhibit all its characters.* 2dly. This alteration may even display a syphilitic character in a subject affected with the disease. 3dly. It may co-exist with symptoms of strangulation, dependent either on stricture of a portion of intestine by the abdominal ring or the indurated and contracted neck of the sac; or by the presence of a loop of intestine in the sac. The strangulation may arise, in this case, from the neck of the sac or the inextensibility of its parietes compressing the displaced parts. Thus Lafay, having reduced an old bubonocele, was obliged, from the continuance of the symptoms, to solicit the relapse of the tumour; and, on opening it, found a loop of intestine strangulated by the thick and indurated parietes of the hernial sac. Lastly, these symptoms may arise from volvulus or other obstruction of the intestinal canal; or sympathetically, from some visceral affection.

To the surgeon, not apprized of the possible existence of exclusive alteration of the sac, diagnosis, then, must be very difficult. The alteration of the sac of crural hernia will exhibit the same characters, but its position and form are modified by the position and form of the hernia itself.

### *Diseases which may be confounded with Alteration of the Hernial Sac, and their Diagnostic Characters.*

I. *Bubonocele.* Precisely resembling bubonocele in its site and figure, the tumour formed by induration of the sac differs from it,—1st, in its volume, which is much smaller than that of hernia; 2dly, in the invariability of its size, except when momentarily augmented by some effort of the patient; 3dly, in its general irreducibility: yet, it is possible, that when the ring is much dilated, and the tumour small, the latter may be in part or wholly returned into the abdomen. Lastly, in the absence of the symptoms of strangulation, if it be uncomplicated, notwithstanding the continuance of the tumour, its long and forcible compression, and evident inflammation. The observations apply equally to the sac of inguinal, and that of crural hernia.

II. *Bubo* can only be confounded with the tumour of the crural sac. The differences are these:—Tumefaction or inflammation of an inguinal gland, whether idiopathic or secondary, is at first small, and successively augments in volume. The hernial tumour, on the contrary, is nearly as large, or quite as extensive, at its commencement, as at any future period. The characters peculiar to these affections will likewise remove much of the doubt. Mistake, in-

* Petit, in his *Maladies Chirurgicales*, tom. ii. p. 380, cites the example of an old and callous hernial sac, remaining in the scrotum after the operation, and becoming subsequently carcinomatous.

deed, would be of little consequence, as both cases require the same treatment.

III. *Varicocele.* The softness and nodosity of the tumour, the blue tinge which it gives to the skin, its diminution or disappearance on pressure, or from the mere effect of the horizontal posture, are the signs which distinguish varicocele from alteration of the inguinal sac, and varix of the vena saphena, at its origin, from alteration of the crural.

IV. The absence of pain, fluctuation perceptible through its evidently thin parietes, and the transparency of the tumour, indicate *encysted hydrocele* of the spermatic cord, and *hydatids* developed in the cord, in the omentum of hernia, or the groin.

V. The softness of a tumour at the ring or crural arch, the previous or concomitant pains at some part of the vertebral column, emaciation and hectic fever, will prove that the tumour is formed by *lumbar abscess.*

VI. *Retraction of the testicle towards the ring*, or its imperfect descent, may be ascertained by the absence of the organ from the scrotum, by the peculiar pain excited on pressure, and the history of the case. The existence of a third testicle may be readily determined by attention to the two latter circumstances.

VII. *Aneurism of the femoral artery*, in its passage beneath the crural arch, is readily distinguishable, at its commencement, from other tumours of this region, and particularly from that formed by the morbid sac of crural hernia. But the firmness of the aneurismal tumour, at an advanced stage, and the obscurity of its pulsations, so embarrass the diagnosis, that many experienced practitioners have been thus led into fatal error. The history of the case can, alone, under these circumstances, elucidate the nature of the tumour.

*Recapitulation.*—1st. The tumour formed by inflammation of the hernial sac, has, at first, nearly the same volume and extent as at any subsequent period. This depends on the simultaneous alteration of all parts of the sac. On the contrary, hydrocele, bubonocele, encysted dropsy of the spermatic cord, and varicocele, exhibit many variations of size, not only from their commencement to their more advanced stages, but from time to time. 2dly. Compression, by even a well-formed bandage, commonly occasions the first; but, on the contrary, prevents the occurrence or relapse of some of the other diseases, as hernia, varicocele, and hydrocele from ascites. Lastly. The first disease necessarily succeeds hernia, while few of the latter are essentially connected with this lesion.

This sketch will probably suffice to illustrate the differences, in character and symptom, of those diseases which exhibit an analogy of situation with the tumour formed by alteration of the hernial sac. A longer detail would have led to numerous repetitions, and to the delineation of objects universally understood.

*Treatment.* The nature of the tumour ascertained, it may, if indolent, be left to itself, without risque, or attempts may be made to resolve it by powerful discutients, as plasters of quicksilver or conium, and especially the solution of gum ammoniacum in vinegar. If there be pain and active inflammation, the latter becomes diffused to the adjacent cellular structure and skin; and fluctuation is perceptible. It is then necessary to accelerate suppuration by emollient remedies. The inflammatory symptoms, when violent, should be moderated by general and local bleeding. The process of suppuration then goes on, and the pus may be allowed to discharge itself spontaneously, or be evacuated by a cutting instrument. If the inflammation be not too active, and suppuration tardy; and particularly, if from the absence of symptoms, it appear that the tumour contains no intestine, the caustic is to be preferred. Thus the activity of the dense and torpid parietes of the sac will be excited, and the irritation will provoke an afflux of the fluids in their substance, and expedite either their resolution or their destruction by the suppurative process. The lapis infernalis, supported and properly confined by diachylon plaster, will answer best. The pus evacuated, stimulant injections may be thrown, or pledgets, spread with digestive, introduced into the cavity of the tumour. Thus, the parietes of the sac will disappear, and a perfect cicatrix be obtained. May not this cicatrix, immediately applied to the opening by which the hernia has protruded, effect its radical cure, by preventing a relapse of it? Richter has seen considerable inflammation induced in the region of the ring by the pad of an inelastic truss, which the patient, in consequence, was obliged to modify. The inflammation terminated in suppuration; and, after the cure of the abscess, the hernia never re-appeared.

If, notwithstanding the use of proper means, the tumour is neither resolved nor inflames, and yet is troublesome from its size, and prevents the application of a truss, excision must be practised. The operation may be performed as in the removal of the thickened sac of strangulated hernia. The tumour being exposed, as in the operation for hernia, should be anteriorly, and in its whole length, slit up. Any existing hernia should be reduced. The pus should then be sponged out; and as much as possible of the parietes of the sac be removed by the bistoury or strong scissars from each margin of the incision. Complete removal would be rendered difficult and dangerous, by the intimate adhesion frequently existing between the spermatic cord and the posterior and internal surface of the tumour. Should the cord lie in front of the tumour, which would be readily ascertained, great caution will be necessary in the incision of the skin and removal of the tumour. The remnant of the sac little retards cicatrization; and must, in fact, consolidate it. By closing the abdominal ring, it will, probably, prevent the recurrence of the hernia: this is a point which future observation and experience must decide.

Were such a tumour invaded by the cancerous action, the whole must be removed without a moment's hesitation, even at the risque of implicating the spermatic vessels and of sacrificing the corresponding testicle.

*Corollaries.*—I. The hernial sac may disappear after reduction of the displaced parts, from adhesion of its parietes kept in contact and irritated by compression of the truss.

II. The acute or chronic inflammation may, on the contrary, produce thickening of the parietes of the sac, and alteration and increase of the fluid exhaled from its internal surface.

III. The characters of the tumour resulting from this alteration, are its site, precisely the same as that of the antecedent hernia; the unchangeableness of its volume; its crepitation on pressure; and elasticity.

IV. Its causes are, a peculiar predisposition; the existence of the hernial sac; the irritation of this sac by compression, strangulation and taxis.

V. The sac of inguinal hernia in the male, and that of crural hernia in the female, must be more frequently the seat of this affection than the sac of other herniæ, because they are more exposed to the mechanical causes of it, and most frequently occur.

VI. The tumour may remain stationary, if there be little or no inflammation; or exhibit the rapid progress of phlegmon, if the inflammation be active.

VII. It may terminate in resolution or suppuration. The pus may escape into the abdomen, and induce violent symptoms.

VIII. It may co-exist, or be complicated, with symptoms of strangulation, or symptoms sympathetically resulting from a morbid state of some of the abdominal viscera.

IX. Its spontaneous opening or its excision is succeeded by a citrix, which may oppose any future displacement.

---

### *Case of Apoplexy.**

——Pinchemaille, aged 38, of sanguine temperament, had, for some years, suffered from slight irritative cough with bloody expectoration, and sense of heat in the breast. These symptoms came on every month by paroxysms, and continued eight or ten days, particularly in the winter. The face and eyes became red during the fit; and the patient complained of violent head-ach, which prevented him from sleepiug, and survived, for several days, the decline of the cough. He was relieved by the use of sedative drinks and application of leeches to the anus. On the 12th of May last, independently of any excess, he was suddenly attacked by great oppression without the power of coughing. Some hours afterward, his tongue became embarrassed, and articulation was soon lost. Towards evening, he grew insensible, and remained lethargic

---

* Observation pour servir à l'Histoire de l'Apoplexie; par M. le Docteur Hébréard. Bulletin de la Faculté de Médecine de Paris, 1816, No. V.

about an hour. An ethereal mixture was administered by the surgeon of the ward; and scarcely had a few spoonfuls been drunk, when a tumour, as large as a fist, and appearing to contain a fluid, arose on the lower and external part of the left fore-arm. The skin was neither painful nor inflamed. At the moment of the appearance of the swelling, the patient regained his senses, and felt no more oppression. On the morrow, I found him in the natural condition; but he was very hungry. Curious to ascertain the nature of the tumour of the arm, and convinced, from its situation, that it was not aneurism, I plunged a bistoury into its centre. A great quantity of bright red frothy blood immediately rushed out, and the tumour sank on its evacuation. The lips of the incision were now brought together by strips of diachylon: it healed in four days, and no trace of the swelling remained. The patient continued tolerably well till the 20th: paroxysms of the cough then recurred with violent head-ache. Ten leeches were applied to the temples without relief. On the 22d, a blister was applied to the nucha. The head-ache was decreased till the 29th, when he was seized with delirium during the night. He got up, and ran screaming about the ward, so that it became necessary to secure him in bed. On the 30th. general debility; face little flushed; the right eye open, and sensible to the light; the left closed by the involuntary depression of the eye-lid, and its pupil much dilated; the left limbs paralysed and insensible. Speech was gone, but he seemed still to hear. The pulse was weak and intermitting. Sinapisms, applied to the feet, produced little redness. Stimulants and cinchona were internally administered. 31st, the same state continued. Blisters applied to the legs. On the following day, death took place in a sort of syncope, without previous rattling.

*Dissection.* The cranial bones were not very firm; the membranes of the brain sound. The organ itself was very soft, particularly in the medullary portion, which, in some parts, precisely resembled cream-cheese. The lateral ventricles were distended by a considerable quantity of albuminous fluid. The lungs were sound, with the exception of some slight adhesions. The abdominal organs presented no unusual appearance. I confess that, from the transudation of arterial blood in the arm, from the periodical exhalation of blood in the interior of the lungs, and particularly from the symptoms of compression of the brain suddenly exhibited by the patient towards the close of life, I expected to find sanguineous effusion in the cranial cavity. May not the phænomena, displayed by the patient, be explained by the soft state of the brain, and by the accumulation of albuminous fluid in the ventricles of the organ?

**Obstetrical.** *Cases of Extra-Uterine Gestation.**

*First Case.* Eve Courad, aged 35, five years married, and in the third month of her first pregnancy, discovered, on Easter Sunday 1808, a painful tumour in the left iliac fossa. Having, one day, fatigued herself with household business, she experienced an attack of fainting. Fifteen days afterwards, in consequence of an extraordinary indulgence in wine, she was seized with vomiting, at the moment of leaving home. It was attended with very violent colic. The abdomen suddenly swelled, and the woman died in a fit of syncope.

The body was examined, under an idea that poison had been administered by the husband. On incision of the abdomen, which was swollen but not discoloured, the intestines were found bathed in blood, part of which was coagulated. This being removed, the intestinal tube was slit open from the cardiac orifice of the stomach to the descending colon, without exhibiting any trace of poison. The coagulated blood, filling the pelvis, was then cleared away; and, something unusual having been detected, the whole of the genital organs were removed. The left Fallopian tube was thus seen forming a tumour, two inches long and nine lines broad; which presented a rupture on the surface corresponding to the pubis. A flocculent structure, speedily recognized for the placenta, just as it appears in the early stages of pregnancy, was interposed between the lips of the wound. On separating these flocculi by immersion in water, the parietes of the tube were found to be half a line in thickness. A transparent membrane was then seen enclosing an embryo; all the parts of which might be readily distinguished. In order to prevent the escape of the embryo, the membrane was preserved entire in alcohol. By incision of the uterus, more voluminous than natural, was verified the assertion of Hunter, that the membrana decidua exists even in cases of extra-uterine gestation. This membrane, soft and pulpy, lined the whole internal surface of the womb. The structure of the organ itself was more soft and vascular, and the ligamenta rotunda thicker, than ordinary. The spermatic vessels seemed enlarged, and the veins were gorged with blood. The left ovary displayed a corpus luteum, and the right, some vesicles filled with transparent lymph. The cervix uteri and vagina were constituted as in women who have never borne children.

*Second Case.* A woman, aged 39, two years after her first delivery by the forceps, again experienced signs of pregnancy. Cessation of the menses, tumefaction of the breasts, vomiting, and pains of the teeth were among the most conspicuous of them. About the end of the third month, a considerable discharge took

---

* Par J. F. Lobstein, M. D. Bulletin de la Société Médicale d'Emulation, No.

place, first of coagulum, next of fluid blood, and lastly of serum. The midwife, on examination, found the uterus about as large as it should be in the fourth month of pregnancy, and its cervix in the natural direction, but more thick and soft than usual. A month after this hæmorrhage, the woman complained of violent pain, gradually increasing, above the right pubis: it was, at first, attributed to indigestion; but, assuming the character of labour, the midwife was summoned on the 16th of August, 1813. She thought, on examining, that she felt obscurely the limbs of a fœtus towards the right side of the uterus, and through the parietes of the vagina. Scarcely had the patient quitted her bed, on a call to the close-stool, when she was attacked with syncope and voided froth by the mouth. The abdomen, at the same time, began to swell, and the face became discoloured; and hence arose a suspicion of internal hæmorrhage. Yet the woman recovered her senses, and no longer complained of pain in the abdomen, but of a burning sensation in the right iliac fossa, gradually ascending to the chest. Dyspnœa soon became urgent, and rendered the horizontal position no longer tolerable. The eyes were fixed and haggard. The saliva flowed slowly from the mouth; and the woman died two hours and a half after the arrival of the midwife, who had never for a moment quitted her.

The body was opened on the same day, in the presence of two other gentlemen. The abdomen, and particularly the pelvis, were full of blood, both fluid and coagulated. After having removed the whole of the genital organs, and cleared them from the coagula with which they were covered, I found, 1st, the uterus preternaturally large, measuring two inches eleven lines, from the os tincæ to the middle of the fundus; two inches nine lines in its greatest breadth; and two inches and a quarter in thickness. The thickness of its parietes was six lines. The cavity of the cervix was filled with concrete mucus, resembling half coagulated white of egg. The os tincæ was perfectly smooth and rounded, with the exception of a fissure on its left side, the relic of the former delivery. The length of the cervix was sixteen lines. The round ligaments were unusually large. The cavity of the uterus exhibited the membrana decidua in a very soft and slender state. The vessels in the substance of the organ were larger than in the unimpregnated condition.—2dly. The right ovary displayed numerous vesicles; some towards the surface, others in the interior of the organ. The largest of them were from one to two lines in diameter. Beneath the external membrane of the ovary was found one corpus luteum, and the traces of a second. The ovary, besides, adhered to the corresponding Fallopian tube. On the surface of the left ovary was a vesicle filled with transparent fluid, coagulable by alcohol. Three other vesicles, containing lymph, and about two lines in diameter, were found in the interior. In the centre of this ovary was discovered a cavity with smooth parietes, and a diameter of five lines in every direction. The membrane constituting the parietes was half a line in thickness: this large vesicle contained a small portion of the fibrine of the blood. One corpus luteum, and the vestiges of three others, were observed in the substance of the ovary.—3dly. The right Fallopian tube was transformed into an egg-shaped tumour, the long diameter of which, directed transversely, was two inches four lines; and the small diameter, twenty lines. At the anterior part of the dilated tube was remarked a rupture, of nineteen lines, presenting the flocculi which constitute the placenta. The parietes of the tube were not every where of the same thickness: they varied from one line and a half to the fourth of a line. The interior of this sac did not appear to be lined by a deciduous membrane: the embryo was distinguished through the transparent membranes of the ovum. It was sixteen lines in length; and its attitude and the direction of its limbs were such as are common to the fœtus of this age. On endeavouring to detach the chorion from the amnios, I found that the former constituted a tolerably strong and dense membrane. The left Fallopian tube presented no unusual appearance.

### PART IV.

### MEDICAL MISCELLANIES.

### THE PORTE FEUILLE, No. V.

OR,

DEPOT FOR DETACHED FACTS AND INTERESTING PHÆNOMENA, OCCURRING IN THE PRACTICE OF THE FRIENDS OF THE MEDICO-CHIRURGICAL JOURNAL.

"Ore trahit quodcunque potest atque addit acervo."

